# Sputum characteristics and airway clearance methods in patients with severe COVID-19

**DOI:** 10.1097/MD.0000000000023257

**Published:** 2020-11-13

**Authors:** Yu Wang, Meng Zhang, Yan Yu, Tao Han, Ji Zhou, Liqing Bi

**Affiliations:** a Department of Neurosurgery Intensive Care Unit; b Department of Critical Care Medicine; c Department of Respiratory and Critical Care Medicine, The First Affiliated Hospital, Nanjing Medical University, Nanjing, PR China.

**Keywords:** COVID-19, expectorant drugs, prone-position drainage, severity, sputum characteristics

## Abstract

Critically ill patients with coronavirus disease 2019 (COVID-19) have a high case fatality rate. Hence, controlling the disease progression of severely ill COVID-19 patients to avoid the development of severe-to-critical COVID-19 is the most important target of COVID-19 treatment. The latest autopsy results of COVID-19 patients have shown the presence of viscous secretions in the airways. However, no studies are available that specifically describe and analyze the sputum characteristics and the effects of various sputum drainage methods on the prognosis of COVID-19 patients. In our study, we found that elderly COVID-19 patients were more susceptible to progression to critical illness (*P* = .024) and were likely to have accompanying lymphopenia (*P* = .035) or increased neutrophil counts (*P* = .019). We observed that there was a higher proportion of patients with Grade 3 sticky sputum in the critically ill group than in the noncritically ill group (*P* = .026), suggesting that changes in sputum characteristics may be one of the early warning signs of critical COVID-19. In addition, we found that the application rates of large doses of ambroxol (*P* = .043) and prone-position drainage (*P* = .037) were relatively high in COVID-19 patients with good prognoses, suggesting that the early application of large doses of expectorant drugs and prone-position drainage in COVID-19 patients may avoid progression to critical illness and improve the prognosis.

## 1 Introduction

An outbreak of a novel coronavirus-induced pneumonia occurred in Wuhan City, Hubei Province, China, in December 2019,^[1]^ and this disease was officially named coronavirus disease 2019 (COVID-19) by the World Health Organization on February 12, 2020. As of April 30, COVID-19 had resulted in 82,874 confirmed cases in mainland China and a total of 4512 deaths nationwide.^[2]^ The illness has become a worldwide pandemic^[3]^ and has overwhelmed health care systems globally.^[4]^ Control of the pandemic situation as quickly as possible and reduction in the case fatality rate have become the greatest challenges worldwide.

According to the 7th edition of guidelines issued by the National Health Commission of the People's Republic of China, COVID-19 cases are categorized into mild, moderate, severe, and critical types.^[5]^ In fact, 90% of the patients who are cured and discharged from the hospital have mild, self-limited cases and the patients heal on their own.^[6]^ The cases that truly determined the death toll involved patients with severe and critical COVID-19. In the relevant existing COVID-19 publications, there is not much information describing critically ill COVID-19 patients. A single-center retrospective study by Yang et al^[7]^ showed that critically ill patients with COVID-19 have a high case fatality rate, showing a 28-day case fatality rate of 61.5%. Hence, controlling the disease progress in severely ill COVID-19 patients to avoid the development of severe-to-critical COVID-19 is the most important target in COVID-19 treatment. In clinical practice, the core link is still to closely monitor the breathing, especially the changes in oxygenation, and heart rate of COVID-19 patients. In addition, the monitoring of related biomarkers is also very important. However, the latest autopsy results of COVID-19 patients have shown many viscous secretions in the airways.^[8]^ Therefore, effective and early removal of mucus in the airway is critical to the prognosis of severely ill COVID-19 patients. To date, no studies are available that specifically describe and analyze the sputum characteristics and the effects of various sputum drainage methods on the prognosis of COVID-19 patients. We participated in the antiepidemic work in Huangshi City, Hubei Province, China, in February 2020 and summarized and analyzed the sputum characteristics and phlegm-dispelling methods used in patients with severe COVID-19 who were hospitalized and treated in a local hospital.

## 2 Methods

### 2.1 Study design and participants

This single-center, retrospective, observational study was performed at Huangshi Central Hospital (Huangshi, China), which is a designated hospital to treat patients with COVID-19. We retrospectively analyzed patients from February 12, 2020, to March 24, 2020, who had been diagnosed with severe COVID-19 at admission, excluding the patients diagnosed with critical COVID-19 that was confirmed at admission. The diagnostic criteria were in accordance with the *Diagnosis and Treatment Plan for COVID-19* (trial edition 7) issued by the National Health Commission of the People's Republic of China.^[5]^ The clinical classification for severe COVID-19 in adults required any of the following criteria: shortness of breath and respiratory rate of ≥30 breaths/minute; oxygen saturation of ≤93% at rest; partial pressure of oxygen in arterial blood (PaO_2_)/concentration of oxygen (FiO_2_) ≤300 mmHg; or pulmonary imaging showing >50% significant progression of pulmonary lesions within 24 to 48 hours. The clinical classification for critical COVID-19 in adult patients was any of the following criteria: presence of respiratory failure requiring mechanical ventilation; shock; or organ failure requiring monitoring and treatment in the intensive care unit.

The identification of severely or critically ill patients was achieved by reviewing and analyzing admission logs and histories from all available electronic medical records and patient care resources. For patients who were alive by March 24, 2020, their status was confirmed on April 7, 2020.

### 2.2 Data collection and evaluation of clinical results

We reviewed clinical electronic medical records, nursing records, laboratory findings, and radiological examination findings for all patients with laboratory-confirmed COVID-19. The admission data of these patients were collected. Data were evaluated and collected by using a case record form. All the patients with severe disease were divided into 2 groups based on whether they changed from severe to critical COVID-19 during hospitalization. We collected data on age, sex, chronic medical histories (hypertension, chronic cardiac disease, chronic pulmonary disease, cerebrovascular disease and diabetes), symptoms from onset to hospital admission (fever, cough or shortness of breath), laboratory values on admission and during hospitalization (white blood cell count, neutrophil count and lymphocyte count), acute physiology and chronic health evaluation (APACHE II) score, disease course and prognosis, treatment (oxygen therapy, antiviral agents, antibiotic agents, airway humidification, expectorant drug usage, nebulized therapy, sputum discharge, and prone-position drainage), and sputum viscosity for each patient on the 5th and 10th day of treatment. As described in previous studies,^[9,10]^ the sputum viscosity scale used in this study had the following 4 categories: Grade 0—no sputum; Grade 1—thin, white foamy sputum that did not remain on the inner wall of the glass connector after sputum suction; Grade 2—moderately viscous sputum that was thicker than Grade 1 sputum, and a small amount of sputum remained on the inner wall of the glass connector after sputum suction although it was easily washed away by water; and Grade 3—severely viscous sputum that was obviously sticky and white or yellow with blood streaks. The sputum suction tube often collapsed due to excessive negative pressure during Grade 3 sputum suction, and the inner wall of the glass connector had a large amount of sputum remaining, which was not easily rinsed with water.

This retrospective study was approved by the Research Ethics Commission of Huangshi Central Hospital, and the requirement for informed consent from study participants was waived by the ethics commission.

### 2.3 Statistical analysis

No hypothesis was made for the present study, so sample size estimation was unavailable, and we included the maximum number of patients who met the inclusion criteria. Data are expressed as the median (range) for continuous variables and the number (%) for categorical variables. Differences between severe cases and severe-to-critical cases were explored using Student *t* test for parametric variables and the χ^2^ test or Fisher exact test for categorical variables. The Statistical Package for Social Sciences (SPSS 20.0) software was used for analysis, and a *P* value <.05 was considered statistically significant.

## 3 Results

During the study period, a total of 41 patients with severe COVID-19 at admission were evaluated, including 25 male (61%) and 16 female (39%) patients with a mean age of 66.7 years (ranging from 32 to 90 years). The patients were divided into 2 groups based on whether they progressed to critical COVID-19 during hospitalization. The average number of days for the progression of severely ill patients to critical illness was 11.6 days. Among the patients who progressed to critical illness, 12 (66.7%) eventually died (Table 1).

**Table 1:** General clinical data, laboratory examinations, and prognosis of 41 severely ill COVID-19 patients.

	Severe cases (n = 23)	Severe-to-critical cases (n = 18)	*P*
Age, y	61.1 (32–81)	71.0 (46–90)	**.024**
Sex			
Male	13 (56.5%)	12 (66.7%)	.509
Underlying diseases			
Hypertension	7 (30.4%)	6 (33.3%)	.843
Diabetes	2 (8.7%)	4 (22.2%)	.224
Heart disease	4 (17.4%)	7 (38.9%)	.123
Chronic obstructive pulmonary disease	1 (4.3%)	1 (5.6%)	.859
Cerebrovascular disease	3 (13.0%)	1 (5.6%)	.423
≥2 underlying diseases	4 (17.4%)	8 (44.4%)	.059
Clinical symptoms and APACHE II score			
Fever	20 (87.0%)	16 (88.9%)	.851
Cough	15 (65.2%)	13 (72.2%)	.632
Difficulty breathing	21 (91.3%)	17 (94.4%)	.702
APACHE II score	13.3 (9–16)	15.1 (12–19)	.647
Laboratory examination results			
WBC count at hospital admission, ×10^9^ cells/L	5.5 (4.2–7.8)	6.1 (4.4–8.3)	.478
Neutrophil count at hospital admission, ×10^9^ cells/L	4.2 (2.8–6.2)	4.7 (2.9–7.4)	.545
Elevated neutrophil count	11 (47.8%)	15 (83.3%)	**.019**
Lymphocyte count at hospital admission, ×10^9^ cells/L	0.7 (0.4–0.9)	0.6 (0.3–0.7)	.638
Reduced lymphocyte count	9 (39.1%)	13 (72.2%)	**.035**
Days between severe and critical illness		11.6 (6–18)	
Fatalities	0	12 (66.7%)	

After the statistical analysis, we found that, compared with severely ill patients, critically ill patients were older (*P* = .024), had more neutrophils (*P* = .019) and lymphopenia (*P* = .035) 7 days after routine treatment (Table 1), and had a higher percentage of Grade 3 sticky sputum (*P* = .026) on the 10^th^ day of routine treatment. The percentages of patients in the critical illness group who received early intravenous administration of large doses of expectorant drugs (≥270 mg ambroxol per day) and drainage in the prone position were lower than those in the severe illness group (*P* = .043, *P* = .037) (Table 2).

**Table 2:** Sputum characteristics and treatment methods of 41 severely ill COVID-19 patients.

	Severe cases (n = 23)	Severe-to-critical cases (n = 18)	*P*
Sputum classification on the 5^th^ day			
0	12 (52.2%)	8 (44.4%)	.623
1	6 (26.1%)	3 (16.7%)	.470
2	4 (17.4%)	3 (16.7%)	.951
3	1 (4.3%)	4 (22.2%)	.083
Sputum classification on the 10^th^ day			
0	4 (17.4%)	1 (5.5%)	.250
1	8 (34.8%)	3 (16.7%)	.194
2	7 (30.4%)	5 (27.8%)	.853
3	4 (17.4%)	9 (50.0%)	**.026**
Inhaled oxygen supplementation			
Nasal cannula	9 (39.1%)	5 (27.8%)	.447
Face mask	2 (8.7%)	2 (11.1%)	.796
High flow	12 (52.2%)	11 (61.1%)	.567
Treatment			
Antiviral therapy	23 (100%)	18 (100%)	NA
Antibiotic therapy	15 (65.2%)	16 (88.9%)	.080
Airway humidification	14 (60.9%)	13 (72.2%)	.447
Nebulized therapy	22 (95.7%)	17 (94.4%)	.859
Large-dose intravenous expectorants	15 (65.2%)	6 (33.3%)	**.043**
Sputum disruption using machine vibration	7 (30.4%)	7 (38.9%)	.571
Clapping on the back of patients to facilitate expectoration	11 (47.8%)	10 (55.6%)	.623
Drainage in the prone position	11 (47.8%)	3 (16.7%)	**.037**

## 4 Discussion

Compared with patients with mild illness, severely ill COVID-19 patients have a poorer prognosis.^[11]^ Once the condition worsens, patients who need mechanical ventilation or have shock and multiple organ failure progress to the critical stage, and they have significantly elevated mortality rates of 67%^[12]^ and even 78%.^[13]^ In this study, among the 41 severely ill COVID-19 patients, 18 progressed to critical illness within 11.6 days on average, indicating a fatality rate of 66.7%. Therefore, early and rapid identification of patients who may progress to critical illness and active intervention in those cases will be the key to improving the survival rate of COVID-19 patients. The potential prognostic factors for patients progressing to critical illness include age,^[14]^ underlying disease,^[7]^ a significant decrease in the lymphocyte count, cytokines and inflammatory markers in peripheral blood, and persistent abnormalities in disseminated intravascular coagulation screening indicators. Close observation of clinical symptoms and regular monitoring of oxygenation indicators in the patients are also key to identifying the deterioration of the patient's condition.^[15]^ This study excluded COVID-19 patients who were already critically ill at hospital admission to analyze the factors that may be related to the progression of severely ill COVID-19 patients. No significant difference in the APACHE II score at admission was found between the 2 groups of patients, suggesting that the initial severity of COVID-19 was similar. After routine treatment according to the current guidelines, elderly patients were more susceptible to progression to critical illness and were likely to have accompanying lymphopenia or increased neutrophil counts, all of which were consistent with previously reported findings.

**Figure 1:**
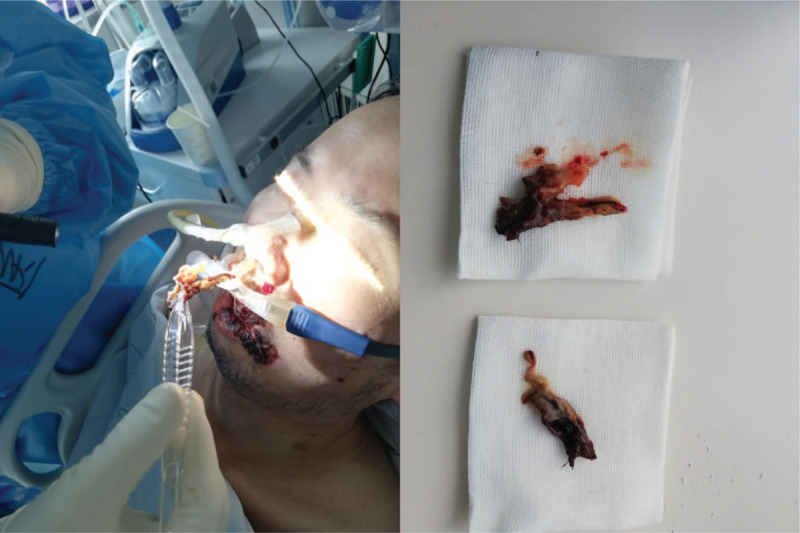
Sticky sputum plugs in the airway of a critically ill COVID-19 patient. COVID-19 = coronavirus disease 2019.

The team of Huazhong University of Science and Technology (Wuhan City, Hubei Province, China) performed an autopsy study of 9 patients with COVID-19 from February 16, 2020, to February 24, 2020. Some results have been published in a Chinese journal.^[8]^ The autopsy results showed a large amount of viscous secretions overflowing from the alveoli in the lungs. In addition, white, foamy mucus, and jelly-like mucus were visible in the bronchial lumen. A previous study, which collected lung tissues by punch necropsy in deceased COVID-19 patients, suggested that the pathological characteristics of COVID-19 are very similar to those caused by severe acute respiratory syndrome (SARS) and Middle East respiratory syndrome coronaviruses, with a more obvious exudative response than in SARS.^[16]^ We also observed sticky sputum plugs in the airway of critically ill COVID-19 patients in this study (Fig. 1). However, COVID-19 mainly manifests with dry cough in the early stage, and sputum is rarely produced. As the disease progresses, sputum may gradually increase, and it is relatively sticky and difficult to expectorate without assistance, thus requiring sputum suctioning. The viscosity of sputum can be measured by a sputum rheometer, but in the clinic, mostly subjective determination is used.^[10,17]^ This study noted that only half of the patients had sputum on the 5^th^ day of admission, and the number of patients with sputum increased on the 10^th^ day of admission. Among them, the proportion of critically ill patients with Grade 3 sticky sputum was higher. Some of the patients had yellow, purulent sputum, which might even occur before the patients progressed from severe to critical illness and was accompanied by an increased neutrophil count and the possibility of a secondary bacterial infection. These findings suggested that changes in sputum characteristics may be one of the early warning signs of the criticality of COVID-19.

To observe the effects of various treatment methods on sputum characteristics and the prognosis of COVID-19 patients, we conducted relevant comparisons and analyses. No significant differences were found in various types of oxygen inhalation, antiviral treatments, and antibacterial treatments in the early stage between the 2 groups of patients with or without progression from severe to critical illness. Before the patient undergoes invasive mechanical ventilation, humidification and nebulized therapy may not be able to effectively act on the lower respiratory tract. The latest consensus on the diagnosis and management of severe COVID-19 ^[18]^ recommends intravenous administration of 300 mg ambroxol daily for airway protection and repair. However, the standard clinical dosage of ambroxol is not uniform, varying from 45 mg to 450 mg/day. The results of this study indicated that the early application rate of high doses of ambroxol in patients who progressed to critical illness is relatively low. However, more research is needed to confirm the role of high-dose ambroxol in sputum removal and airway protection in COVID-19 patients. According to the guidelines on respiratory rehabilitation for patients with COVID-19,^[19]^ techniques, such as postural drainage, clapping on the back of patients to facilitate expectoration, and sputum disruption with machine vibration, can be used to improve sputum retention and difficulty in sputum expectoration among patients. In addition, the pathophysiological characteristic of acute respiratory distress syndrome (ARDS) due to severe and critical COVID-19 is the heterogeneity of lung injury. Prone-position ventilation is a very important rescue therapy for critically ill COVID-19 patients.^[20]^ This study did not show any differences in the application of machine or manual sputum removal in the 2 groups of severe and critically ill COVID-19 patients. However, as a sputum drainage measure, prone positioning has been applied in COVID-19 patients without mechanical ventilation in clinical practice. The application rate of prone-position drainage is relatively high in COVID-19 patients with good prognosis, suggesting that early application of prone-position drainage in COVID-19 patients may avoid the progression to critical illness and improve prognosis.

This single-center retrospective observational study had a relatively small sample size, and the observation indicators were not comprehensive. The retention of sputum and the determination of sputum characteristics in this study were not in accordance with the unified and objective standard, which needs further verification in a large-scale study combined with more autopsy results of COVID-19 patients.

## 5 Conclusions

The mortality of critically ill patients with COVID-19 is considerably high, and changes in sputum characteristics may be one of the early warning signs of the criticality of COVID-19. The early application of high doses of ambroxol and prone positioning in COVID-19 patients may avoid progression from severe to critical illness and improve patients’ prognosis.

## Acknowledgments

The authors thank all patients and their families involved in the study.

## Author contributions

Yu Wang, data collection and writing; Meng Zhang, data collection; Yan Yu, data collection; Tao Han, data collection and analysis; Ji Zhou, data analysis; Liqing Bi, study design and writing

**Conceptualization:** Liqing Bi.

**Data curation:** Yu Wang, Meng Zhang, Yan Yu, Tao Han.

**Formal analysis:** Tao Han, Ji Zhou.

**Writing – original draft:** Yu Wang.

**Writing – review & editing:** Liqing Bi.

## References

[1] Huang C Wang Y Li X Clinical features of patients infected with 2019 novel coronavirus in Wuhan, China. Lancet 2020;395:497–506. 31986264 10.1016/S0140-6736(20)30183-5 PMC7159299

[2] National Health Commission of the People's Republic of China. Novel coronavirus in China. Available at: http://www.nhc.gov.cn/xcs/yqtb/202005/11f6b5e28be64f28b5b84eed2984ed60.shtml Updated April 30, 2020.

[3] Cucinotta D Vanelli M WHO declares COVID-19 a pandemic. Acta Biomed 2020;91:157–60. 32191675 10.23750/abm.v91i1.9397 PMC7569573

[4] Fauci AS Lane HC Redfield RR Covid-19- navigating the uncharted. N Engl J Med 2020;382:1268–9. 32109011 10.1056/NEJMe2002387 PMC7121221

[5] National Health Commission of the People's Republic of China. Diagnosis and treatment protocols of pneumonia caused by a novel coronavirus (trial version 7). J Cardiovasc Pulmon Dis 2020;39:103–7.

[6] Guan WJ Ni ZY Hu Y Clinical characteristics of Coronavirus Disease 2019 in China. N Engl J Med 2020;382:1708–20. 32109013 10.1056/NEJMoa2002032 PMC7092819

[7] Yang X Yu Y Xu J Clinical course and outcomes of critically ill patients with SARS-CoV-2 pneumonia in Wuhan, China: a single-centered, retrospective, observational study. Lancet Respir Med 2020;[Epub ahead of print]. 10.1016/S2213-2600(20)30079-5 PMC7102538 32105632

[8] Liu Q Wang RS Qu GQ Gross examination report of a COVID-19 death autopsy. Fa Yi Xue Za Zhi 2020;36:21–3. 32198987 10.12116/j.issn.1004-5619.2020.01.005

[9] Shen MF Zhang HY Study on the optimal suctioning negative pressure based on sputum viscosity in brain-injured patients with tracheotomy. Chin J Nurs 2009;44:694–7.

[10] Zanasi A Mazzolini M Tursi F Homeopathic medicine for acute cough in upper respiratory tract infections and acute bronchitis: a randomized, double-blind, placebo-controlled trial. Pulm Pharmacol Ther 2014;27:102–8. 23714686 10.1016/j.pupt.2013.05.007

[11] Wang C Horby PW Hayden FG A novel coronavirus outbreak of global health concern. Lancet 2020;395:470–3. 31986257 10.1016/S0140-6736(20)30185-9 PMC7135038

[12] Arentz M Yim E Klaff L Characteristics and outcomes of 21 critically ill patients with COVID-19 in Washington state. JAMA 2020;[Epub ahead of print]. 10.1001/jama.2020.4326 PMC7082763 32191259

[13] Zhou F Yu T Du R Clinical course and risk factors for mortality of adult inpatients with COVID-19 in Wuhan, China: a retrospective cohort study. Lancet 2020;395:1054–62. 32171076 10.1016/S0140-6736(20)30566-3 PMC7270627

[14] Liu K Chen Y Lin R Clinical features of COVID-19 in elderly patients: A comparison with young and middle-aged patients. J Infect 2020;80:e14–8. 10.1016/j.jinf.2020.03.005 PMC7102640 32171866

[15] Li HC Ma J Zhang H Thoughts and practice on the treatment of severe and critical new coronavirus pneumonia. Chin J Tuberc Respir Dis 2020;43:E038. 10.3760/cma.j.cn112147-20200312-00320 32186172

[16] Xu Z Shi L Wang Y Pathological findings of COVID-19 associated with acute respiratory distress syndrome. Lancet Respir Med 2020;8:420–2. 32085846 10.1016/S2213-2600(20)30076-X PMC7164771

[17] Zhong L Xiong Y Zheng Z Effect of short-term inhalation of warm saline atomised gas on patients with non-cystic fibrosis bronchiectasis. ERJ Open Res 2020;6:10.1183/23120541.00130-2019 PMC7008135 32055629

[18] COVID - 19 Treatment Cooperation Group in Tongji Hospital Wuhan. Diagnosis and treatment protocols of severe pneumonia caused by a novel coronavirus. J Intern Intens Med 2020;26:1–5.

[19] Wang C Xie YX Zhao HM Recommendations for respiratory rehabilitation of coronavirus disease 2019 in adult. Chin J Tuberc Respir Dis 2020;43:308–14. 10.3760/cma.j.cn112147-20200228-00206 32294814

[20] Pan C Chen L Lu C Lung recruitability in SARS-CoV-2 associated acute respiratory distress syndrome: a single-center, observational study. Am J Respir Crit Care Med 2020;201:1294–7. 32200645 10.1164/rccm.202003-0527LE PMC7233342

